# Discovering immunoreceptor coupling and organization motifs

**DOI:** 10.3389/fimmu.2023.1253412

**Published:** 2023-09-04

**Authors:** Michael Reth

**Affiliations:** ^1^ Department of Molecular Immunology, Biology III, Faculty of Biology, University of Freiburg, Freiburg, Germany; ^2^ Signaling Research Centers CIBSS and BIOSS, University of Freiburg, Freiburg, Germany

**Keywords:** antigen receptors, ITAM, nanoscale receptor organization, leucine zipper, resting state of lymphocytes

## Abstract

The recently determined cryo-EM structures of the T cell antigen receptor (TCR) and B cell antigen receptor (BCR) show in molecular details the interactions of the ligand-binding part with the signaling subunits but they do not reveal the signaling mechanism of these antigen receptors. Without knowing the molecular basis of antigen sensing by these receptors, a rational design of optimal vaccines is not possible. The existence of conserved amino acids (AAs) that are not involved in the subunit interaction suggests that antigen receptors form higher complexes and/or have lateral interactors that control their activity. Here, I describe evolutionary conserved leucine zipper (LZ) motifs within the transmembrane domains (TMD) of antigen and coreceptor components that are likely to be involved in the oligomerization and lateral interaction of antigen receptor complexes on T and B cells. These immunoreceptor coupling and organization motifs (ICOMs) are also found within the TMDs of other important receptor types and viral envelope proteins. This discovery suggests that antigen receptors do not function as isolated entities but rather as part of an ICOM-based interactome that controls their nanoscale organization on resting cells and their dynamic remodeling on activated lymphocytes.

## Introduction

Over the past three years the human population has experienced a major viral pandemic with the accompanying loss of life and economic decline. What will hopefully now end this health crisis is the establishment of an adaptive T and B cell immunity against the pandemic SARS-CoV-2 virus and its mutants in vaccinated and/or infected individuals. The antigen receptors on T and B cells play an essential role in adaptive immunity. The T-cell antigen receptor (TCR) and the B-cell antigen receptor (BCR) have a similar molecular design. They are multi-protein complexes consisting of a ligand-binding part and a signaling subunit. For the αβTCR, these are the α/β chains binding to peptide-loaded MHC (pMHC) molecules and the CD3/zeta signaling complex consisting of CD3γ/CD3ε and CD3δ/CD3ε heterodimers and a TCR-ζ/TCR-ζ homodimer (1).

For the BCR, the ligand-binding part consists of the different classes of the membrane-bound immunoglobulin (mIg) and the Igα/Igβ (CD79a/CD79b) heterodimer (2). All signaling subunits of these receptors carry a dual tyrosine motif in their cytoplasmic tail, which I first described in 1989 (3) and, which became known as the immunoreceptor tyrosine-based activation motif (ITAM). It has been shown that the two ITAM tyrosines, once phosphorylated, are engaged by the dual SH2 domains of ZAP-70 and Syk on the TCR and BCR, respectively (4, 5). The structural details how ITAM phosphorylation is prevented in resting T and B cells and how exactly antigen binding to the receptors results in increased accessibility of the ITAM tyrosines for phosphorylation and kinase binding is not known at present. One part of this regulation is a a shift in the kinase/phosphatase equilibrium at resting and activated antigen receptors. Another part is the nanoscale organization of the antigen receptors on the plasma membrane that seems to be essential for their regulation (6–8). On resting B cells, the IgM-class (IgM-BCR) and IgD-class (IgD-BCR) BCR reside within separated nanoclusters of different protein/lipid composition and with different topological features (2). The IgM-BCR is localized in a non-raft lipid domain and forms clusters that are associated with curved membranes on a network of ridges and the tip of microvilli (9). In contrast, the IgD-BCR is found associated with CD19, CD81, CD20, CXCR4, BAFFR and CD40 within a raft-type lipid nano-domain that also harbors GM1. For the stability of the IgD-type nanoclusters, CD20 is required and within the clusters, the different receptors are also functionally connected (10). For example, CXCR4 signaling is defective on the surface of IgD-BCR-deficient murine B cells (11).

Antigen-dependent activation of B cells results in a nanoscale reorganization of antigen receptors and their membrane environment. As proposed by the dissociation activation model (DAM), the IgM-BCR clusters are opened and gain access to the coreceptor CD19 and to CD20 (10, 12). How nanoscale receptor clusters are established and what molecular events are involved in their remodeling are currently unknown. I suggest here that not only the extracellular or intracellular parts of immunoreceptors, but also their TMD play an important role in their proper function. This suggestion is supported by the discovery of sequence motifs within these TMDs that are likely to regulate the nanoscale organization of these receptors on resting and their dynamic reorganization on activated lymphocytes.

## Results

### The asymmetric organization of the BCR and TCR complexes

In most immunology textbooks, TCR- and BCR complexes are depicted as symmetric structures with a signaling heterodimer placed on each side of the ligand-binding parts. Based on the fact that the mIgM molecule is a symmetric homodimer, we initially also proposed a symmetric model for the IgM-class BCR (IgM-BCR) complex, in which the mIgM molecule binds two CD79a/CD79b heterodimers (13). However, biochemical analysis has shown that the mIgM molecule and the CD79a/CD79b heterodimer form a 1:1 complex rather than a 1:2 complex (14). The now resolved cryo-EM structures confirm the 1:1 model of the IgM-BCR complex and show an asymmetric assembly between the ligand-binding and signaling part of these antigen receptors (15–17). Specifically, in the murine IgM-BCR structure the CD79a/CD79b heterodimer predominantly interacts with only one of the two heavy chains (μHC and μHC’) of the mIgM molecule (17). The monomeric αβTCR also shows an asymmetric organization with the CD3 complex occupying only one side of the αβTCR (18). Interestingly, the cryo-EM structures of the pMHC-bound or free αβTCR do not reveal major alterations upon ligand binding (19). Coupling between the antigen receptor components occurs via three distinct contact sites, namely the membrane proximal Ig domains, the connecting peptides and the TMDs, with the latter two playing a dominant role in complex formation. The TMDs of all antigen receptor components are single-spanning alpha-helixes, covering a space within the membrane that has been previously underestimated and drawn much too small in many schematic representations of antigen receptor organization. Thus, only the recent cryo-EM structures highlight the importance of the TMDs for the formation and stability of antigen receptor complexes.

### Analysis of the lateral accessibility of the monomeric IgM-BCR complex

**Figure 1:**
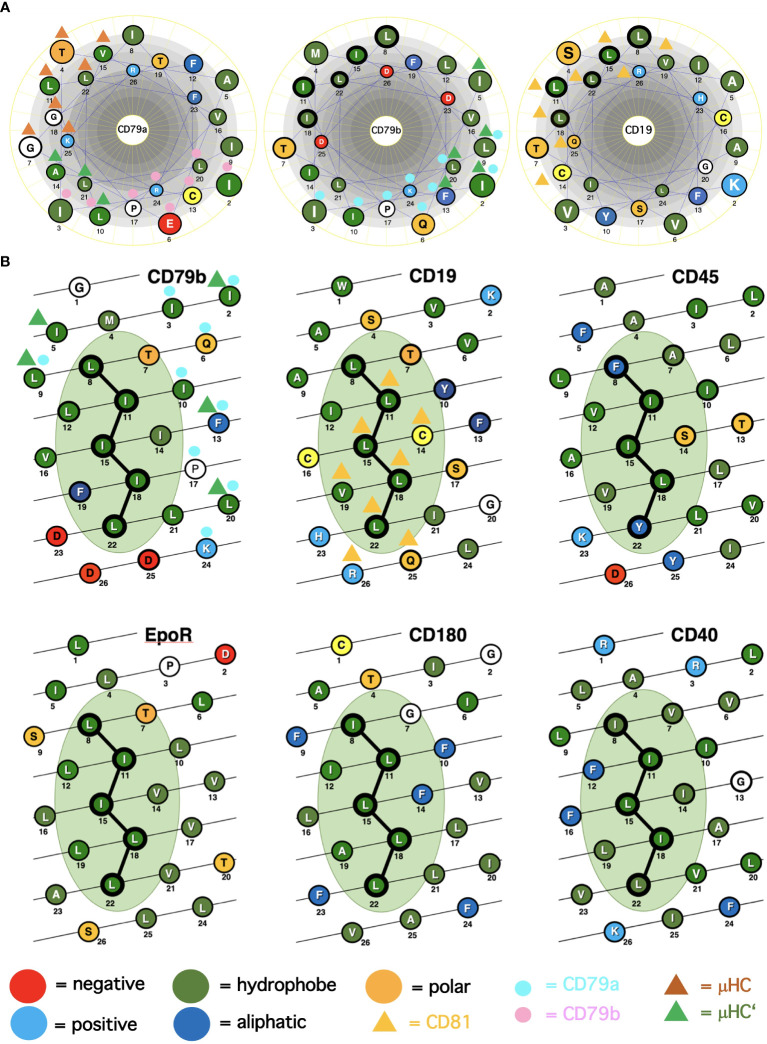
Alpha-helical schemes applied for the ICOM discovery. **(A)** Comparison of the TMD AAs sequences (single-letter code) of the BCR signaling subunits CD79a, CD79b and of the coreceptor CD19 represented as helical wheel. The AAs are color coded according to their chemical features, as indicated. Those AAs involved in BCR or CD19/CD81 complex formation are marked by colored triangles and circles. The ICOM AAs which form the central LZ-structure are outlined by a bold circle. **(B)** Comparison of the TMD AAs sequences (single-letter code) of ICOM-positive membrane proteins represented as alpha-helical barrel. The AAs are color coded according to their chemical features. Those AAs involved in BCR or CD19/CD81 complex formation are marked by colored triangles and circles. The ICOM AAs are connected by a line and outlined by a bold circle.

A major question arising from the known cryo-EM structures is why the basic structure of the antigen receptor complexes is asymmetric. A possible answer to this question is that the asymmetric organization allows these receptors to interact on the lymphocyte membrane either with themselves (forming dimers and oligomers) or with coreceptor modules that regulate or amplify the signal transduction of the antigen receptors. Following this idea, I took a closer look at the conserved TMD AAs that are either engaged in the formation of the 4-alpha-helical bundle of the IgM-BCR TMDs or exposed to the lipid bilayer. The TMDs of the CD79a and CD79b signaling component have different positions in the IgM-BCR structure. The CD79a TMD binds on one side of the alpha helix to the CD79b TMD and on the other side to the TMDs of the μHC and μHC’ (**Figure 1A**). Indeed, it is predominantly CD79a that couples the CD79a/CD79b heterodimer to the mIgM molecule. The CD79b TMD is only engaged on one side in the binding to the CD79a TMD and, to a lesser extent, to the μHC’ (**Figure 1**). The exposed, free side of the CD79b TMD carries either a leucine or isoleucine residue at the TMD position 8, 11, 15, 18, 22, which are part of a leucine zipper (LZ) heptad motif involved in the interaction of different TMD alpha-helixes (20). Within a heptad (**a**bc**d**efg) motif, the a- and d-position residues form the hydrophobic core of the interface between the interacting alpha helices.

### Discovery of a potential lateral interaction motif with the TMDs of immunoreceptors

From our previous studies of the BCR conformation and interaction on the B cell membrane using the Fab-based proximity ligation assays (Fab-PLA), we learned that the IgM-BCR established a close contact with the CD19 coreceptor upon B cell activation (6). A look at the AAs composition of the human CD19 TMD revealed a series of 5 leucine residues located on one side of the CD19 TMD alpha helix at the same position as the LZ components of the CD79b TMD (**Figure 1A**). It is thus likely that the LZ structure within the CD19 TMD is involved in the heterodimerization of the IgM-BCR with the CD19 coreceptor. Interestingly, in our Fab-PLA studies, we detected the IgM-BCR/CD19 interaction only on activated but not on resting B cells. The reason for this restriction may be the finding that CD19 is only present on the B cell surface in association with the CD81 protein (21). Based on a recent cryo-EM study, a model of the CD19/CD81 complex can be drawn. Although the present CD19/CD81 cryo-EM is of low resolution, it shows that the LZ structure within the CD19 TMD is covered by one of the TMD alpha-helixes of CD81 and one can predict that the LZ residues are not available for binding to the CD79b TMD (22). However, B cell activation appears to be accompanied by the dissociation of the CD19/CD81 complex, releasing CD19 for the potential TMD interaction with the IgM-BCR complex (23). As explained above, the association between the ligand-binding and the signaling subunits of the IgM-BCR involves the extracellular Ig domains and the connecting peptides in addition to the TMDs. Thus, it is feasible that not only the LZ structure within the TMDs but also additional sites take part in the CD79b/CD19 complex formation. One of these sites seems to be the juxtamembrane region which contains a series of complementary positively (H23,Q25,R26) and negatively (D23,D25,D26) charged AAs in the CD19 and CD79b AA sequence, respectively (**Figure 1A**). An alignment of the human TMD sequences of CD79b and CD19 according to the found LZ structure shows that these two sequences carry 9 identical or similar AAs (**Table 1**). Thus, I could define an LZ-containing A-motif: TLxx(LI)(LI)xx(LI)xx(LI)xx(LI)L, which is shared by both proteins and that may play an important role in the organization of antigen receptors and their coupling to lateral interactors. For this and related LZ motifs (see below), I propose the name “immunoreceptor organization and coupling motifs” (ICOMs).

**Table 1:** ICOMs motif searches related to the CD79b and CD19 TMD sequence.

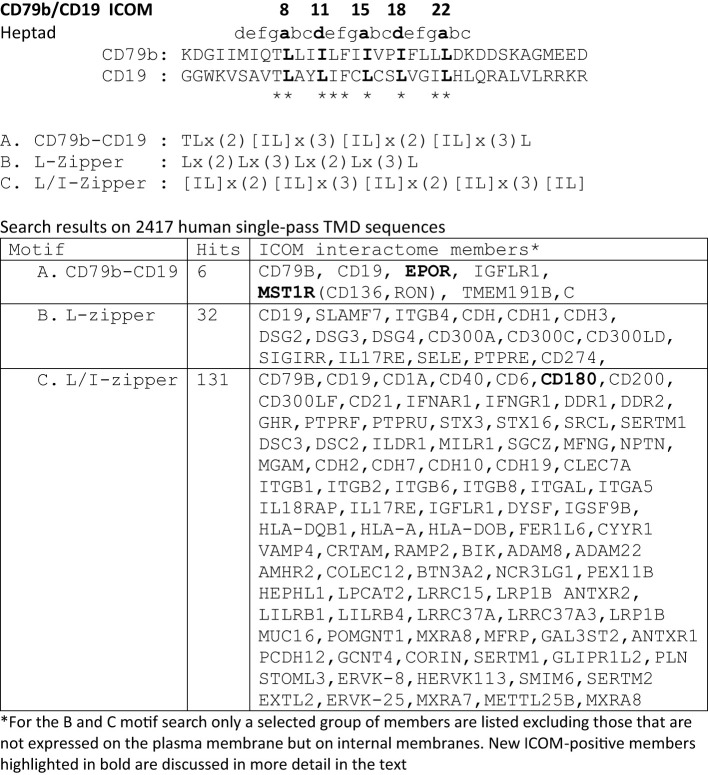

### Motif search for ICOM-family members

A prosite motif search of the TMD + 5 AA flanking sequences of 2417 single-pass transmembrane proteins extracted from the human uniprot database (TMD5) for the defined A-motif yielded only 6 hits (**Table 1**). In addition to CD79b and CD19, the A-motif is found in the TMD sequence of the erythropoietin receptor (EpoR), the insulin growth factor-like receptor 1 (IGFLR1), the macrophage stimulating 1 receptor (MST1R), also known as CD136 or RON, and the transmembrane proteins TMEM191 of unknown function. The result of the A-motif search was verified by a TMD sequence comparison according to the 5 positions of the heptad LZ motif (**Table 2**). A problem that arises with such a comparison is that the start of a TMD sequence is not always well defined and that the length of this sequence can differ between 19-24 AAs. In my TMD sequence comparison, I aim to place the first heptad repeat AA at (or near) the position 8 and the last at position 22, which should be preferentially followed in the next 1-4 positions by a charged or polar AA. With such an alignment the 5 positions of the heptad repeat are occupying a central space within the alpha-helical barrel representation of a TMD sequence (**Figure 1B**). Of the 6 hits from the A-motif search, only the MST1R TMD caused a problem and allowed alternative ways of LZ motif sequence alignment. One should keep in mind that an LZ sequence is a rather flexible dimerization structure that is not restricted to leucine or isoleucine residues. Indeed, 1 or 2 of the 5 heptad repeat positions of an LZ structure could carry other AAs than leucine or isoleucine, such as hydrophobic or polar, without compromising the dimerization function (24–26). Cells appear to exploit this binding flexibility to either increase or decrease the affinity of an ICOM : ICOM interaction (see below).

**Table 2:** Selected TMD sequences from the ICOM A-C motif searches.

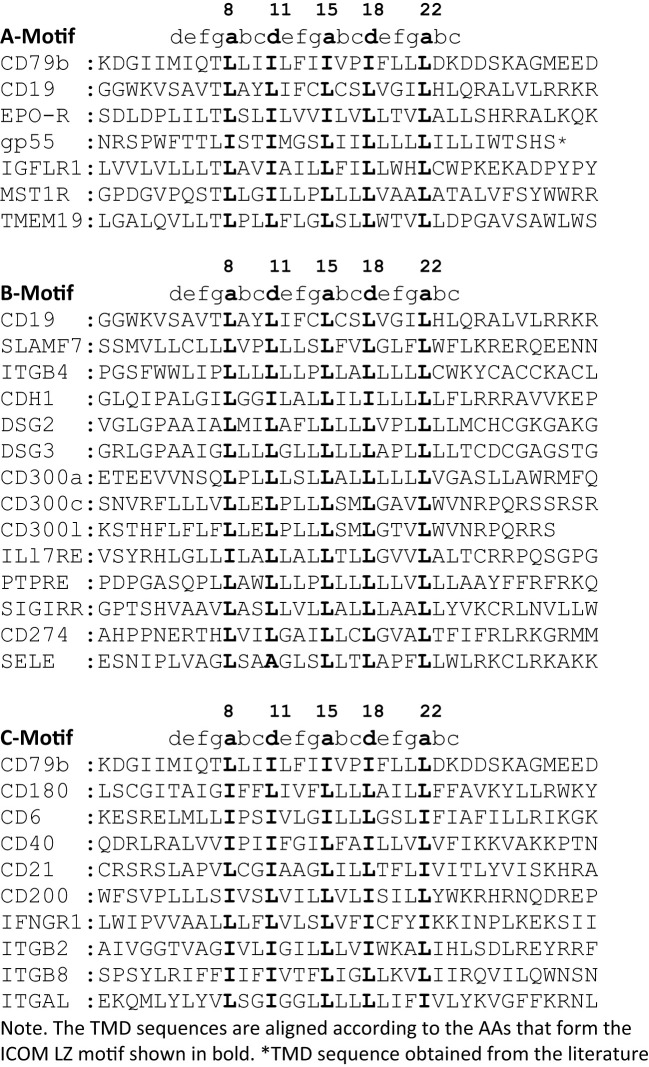

Next, I conducted a prosite search for a leucine-only LZ motif (B motif): Lx(2)Lx(3)Lx(2)Lx(3)L found in the CD19 TMD. This search yielded 32 hits, including several adhesion proteins such as cadherins (CDH, CDH1, CDH3), desmogleins (DSG, DSG2, DSG4) and the integrin ITGB4. It is thus feasible that an ICOM-based interaction between adhesion and antigen receptors is involved in the activation of lymphocytes in contact with cell-bound antigens.

A prosite search for ICOM members with a mixed leucine/isoleucine LZ motif (C-motif): [IL]x(2)[IL]x(3)[IL]x(2)[IL]x(3)[IL] identified 131 membrane proteins. This search included the 32 MPs identified in the B-motif search and extended the group of adhesion proteins carrying an ICOM within their TMD. Interestingly, the identified ICOM-positive members expressed on B cells are CD40, CD180 also known as RP105 (see below). Furthermore, I identified the scavenger receptor family member CD6, which fine-tunes TCR signaling, as an ICOM-positive protein.

### The EpoR/p55/sf-Stk story

The finding that the TMD of EpoR contains a LZ structure similar to that of CD79b and CD19 is of particular interest since this receptor has been extensively studied over the last 20 years. Like the BCR, the EpoR was once thought to be activated either by dimerization of two monomers or by an EPO-dependent conformational change that brings two TMDs into close proximity to each other (27). However, this idea has been challenged by studies showing that the resting EpoR already forms a dimer and that the LZ motif within its TMD is involved in receptor dimerization (28, 29). The EpoR can be activated not only by the binding of its ligand EPO but also by coexpression of the envelope-related glycoprotein gp55 of the Friend spleen focus forming virus (F-SFFV), which causes erythroblastosis in an EpoR-dependent manner (30). Interestingly, the TMD of gp55 carries an ICOMs-related LZ motif (**Table 2**). Newer data show that the TMD of gp55 either forms homodimers or binds to the TMD of EpoR (31). Thus, dissociation of the TMDs of the EpoR dimer either by Epo or by gp55 has been discussed as the activation mechanism of this receptor. Recent studies suggest that in addition to EpoR and gp55 also a third player, namely the tyrosine kinase sf-Stk, is required for fibroblast transformation by F-SFFV by forming a tripartite EpoR/p55/sf-Stk complex as an oncogenic driver (32, 33). Interestingly, sf-Stk is an N-terminal truncated form of murine MST1R/RON, which is one of the six hits of the A-motif search of the human membrane proteome. Thus, three different ICOM-positive membrane proteins are implicated in oncogenic complex formation and cellular transformation. In summary, genetic and biochemical studies support the notion that ICOM-carrying TMDs are involved in the homo- and/or heterodimerization of membrane proteins and regulate important biological processes such as cellular transformation.

### The RP105/CD180 story

**Figure 2:**
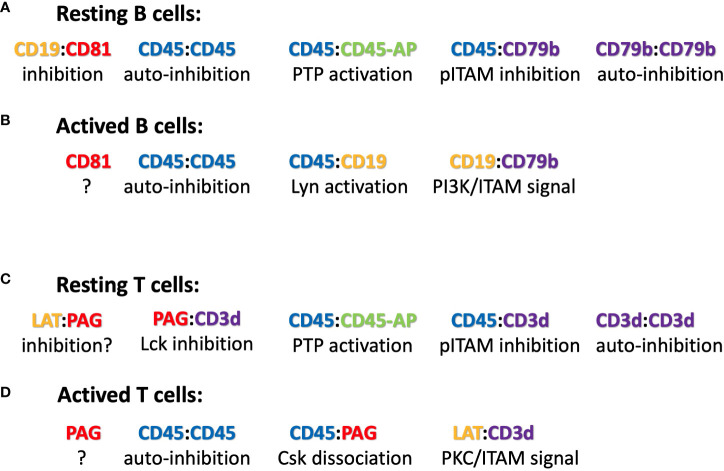
Suggested ICOM-based TMD interactions of membrane proteins on **(A)** resting and **(B)** activated B cells as well as on **(C)** resting and **(D)** activated T cells. A possible regulatory or signaling function of the depicted ICOM : ICOM complex is indicated below each suggested interaction.

The radio-protective 105 (RP105) antigen was discovered in a screen for monoclonal antibodies (mAb) that protect B cells from X-ray irradiation induced apoptosis. Surprisingly, B cells exposed to the RP105 mAb underwent a massive mitogenic B cell expansion and differentiation (34). The cloning and sequencing of RP105, now known as CD180, identified this protein as a new member of the toll-like receptor family (35). CD180 is an orphan receptor that forms a dimer and can interact with toll-like receptor 4 (TLR4). Similar to TLR4 which requires binding to MD-2, CD180 reaches the cell surface only in association with the adaptor MD-1 (36). It remained a puzzle that the mitogenic signaling via CD180 did not involve the classical Toll-like/Myd88 signaling pathway but rather depended on the components of the BCR signaling pathway such as BTK and Lyn (37, 38). My discovery that the TMD of CD180 carries an ICOM provides a solution to this puzzle (**Figure 2**). I suggest that on resting B cells, CD180 is part of the inhibitory IgD-type nanocluster. Upon exposure of B cells to either cognate antigen or to anti-RP105 mAb the inhibitory complex is resolved and CD180 becomes part of the ICOM interactome on activated B cells that promotes CD19/IgM-BCR complex formation and ITAM/PI-3K signaling. The finding that the extracellular part of the CD180/MD-1 complex can bind to the TLR4/MD-2 complex (39) and my suggestion that the TMD of CD180 is associated with the ICOM interactome suggest a direct link between the innate and adaptive immune system. This suggestion also provides an explanation for the cooperation between TLR4 and BCR in B cell activation (40, 41).

### Viral ICOMs and their targets

When I discovered the dual tyrosine motif (now known as ITAM) in the cytoplasmic tail of the signaling components of the BCR and TCR, as well as Fc receptors, I noticed that the glycoprotein gp30 of the bovine leukemia virus (BLV) also carried such a motif (3). Subsequently, other ITAM-bearing viral proteins were described, demonstrating that viruses employ the ITAM-dependent signaling modules as part of their propagation strategy (42).

Given the importance of the ICOM interactions for lymphocyte regulation and activation, it is likely that viruses and tumor cells employ the ICOM coupling and organization function. Indeed, I found that BLVgp30 carries not only an ITAM in its cytoplasmic tail but also an ICOM within the TMD sequence (**Table 3**). BLVgp30 may thus interact with CD19 in an ICOM-dependent manner to establish an ITAM/PI-3K signaling axis and this might be one of the drivers of bovine leukemia. Whether or not BLVgp30 or the env proteins of related viruses can also directly interact with the antigen receptors in the way that gp55 activates the EpoR remains to be investigated. Clearly, a deregulated ICOM interaction is involved not only in viral infection but also in oncogenic transformation. Another example for this is the platelet-derived growth factor receptor beta (PDGFRb), whose TMD can form ligand-independent homodimers (43) and interact with the bovine papillomavirus BPVE5R transmembrane protein (44, 45). The TMD of both proteins carries an ICOM-related LZ structure, which may promote their interaction and tumor development (**Table 3**).

**Table 3:** TMD Sequences of ICOM-positive Receptor Components.

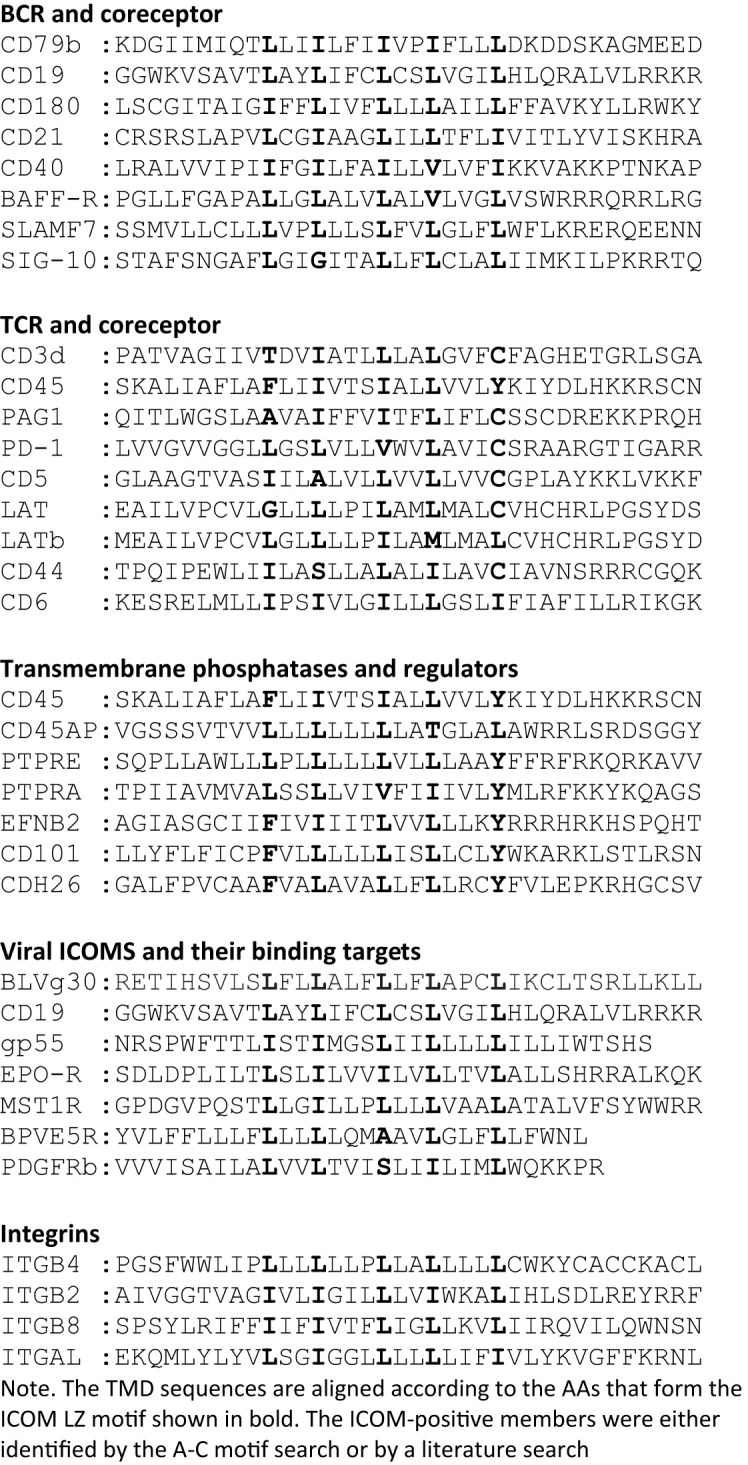

### The CD45 ICOM-related TMD sequence

In our nanoscale Fab-PLA studies, we found that the transmembrane phosphatase CD45 (PTPRC) is associated with the IgM-BCR complex on resting but not on active B cells. By dephosphorylating the two ITAM tyrosines, CD45 has a negative signaling role as a gatekeeper of IgM-BCR activation (8). However, upon B cell activation, CD45 dephosphorylates the negative regulatory pY593 of Lyn and has a positive role in IgM-BCR signal amplification. Thus, like many signaling proteins, CD45 can alter its signaling functions depending on its location and association. To learn more about the molecular association of CD45 with the resting IgM-BCR, I had a look at the evolutionary highly conserved TMD sequence of CD45 and found that one side of the CD45 TMD carries an ICOM-related sequence in which the 2 outer AAs of the 5 LZ positions are occupied by a phenylalanine and tyrosine instead of a leucine (**Figure 1B**). When evaluating the CD45 ICOM, it should be kept in mind that within the lipid bilayer, a phenylalanine or tyrosine can form tight complexes with either a leucine or isoleucine residue (46). This notion is supported by the described AAs interactions between the TMDs in the BCR complex (17). A CD45 ICOM-related sequence has also been found in the TMD of human CD101, also known as immunoglobulin superfamily member 2 (IGSF2) and cadherin 26. A related sequence is also present in the TMD of two further human PTPRs namely PTPRE and PTPRA (**Table 3**).

It has been suggested that CD45 and the related transmembrane phosphatase PTPRA can form dimers and that dimerization is mediated by the extracellular domain and the TMDs of these phosphatases. It is therefore possible that the ICOM of these phosphatases is involved in their dimerization. The CD45 dimer is less active and seems to be the autoinhibited form of this tyrosine phosphatase (47). Thus, dissociation and reorganization of the CD45 dimer appears to be associated with an increased enzymatic activity of CD45. Interestingly this process is regulated by the CD45-associated protein (CD45-AP or PTPRC-AP), a small membrane protein that binds to CD45 via its leucine-rich TMD (48). Closer examination of the CD45-AP TMD sequence reveals that it carries an ICOM-related LZ structure (**Table 3**). T cell studies have shown that the binding of CD45-AP to CD45 not only leads to the dissociation of the CD45 dimer, but also to an increased phosphatase activity and CD45/TCR interaction (49). In this respect, the ICOM-mediated CD45/CD45-AP interaction is similar to that of the EpoR/gp55 pair. Thus, on resting B cells, CD45-AP may promote CD45:CD45 dissociation and the binding of CD45 to CD79b. In this way, an active tyrosine phosphatase is coupled to the IgM-BCR and this association may help to maintain the resting state of B lymphocytes.

### Model of an ICOM-based dynamic regulation of B cell maintenance and activation

The discovery of conserved ICOMs within the TMD of one of the two BCR signaling components and several BCR co-receptors suggests that B cell maintenance and activation is a very dynamic process involving different ICOM interactions. Based on this new information, one could imagine the following scenario for the regulation and activation of B cells via specific TMD : TMD interactions (**Figure 2**). On the surface of resting B cells, the IgM-BCR may form several inhibitory complexes that prevent deregulated signaling. One complex is an autoinhibitory IgM-BCR oligomer that is maintained by an ICOM-dependent CD79b/CD79b and a HC:HC homodimerization (50). Within the closed IgM-BCR oligomer, the CD79b ICOMs are not accessible for interaction with CD19 or other positive signaling components. A second inhibitory complex could be formed via the ICOM-dependent interaction of the monomeric IgM-BCR with the CD45/CD45-AP complex as discussed above. In contrast to that of the IgM-BCR, the IgD-BCR ICOM interactome is located in a raft lipid domain and may be organized by a CD20/CD81/CD19 tetraspanin web (model not shown). Once an ICOM : ICOM-negative regulatory complex is formed and delivered to the plasma membrane, it could be further stabilized by extracellular cross-linkers. A likely candidate for this function is galectin-9 (Gal9), a dimeric adaptor protein carrying two carbohydrate recognition domains separated by a flexible linker. Exposure of B cells to Gal9 suppresses IgM-BCR mobility and signaling (51). Gal9-deficient B cells are hyperactive, thus demonstrating the negative regulatory role of this lectin. Interestingly, several of the identified binding targets of Gal9 such as CD45, CD79b and CD180 also carry an ICOM with in their TMD. Upon B cell activation these ICOM complexes may be remodeled as outlined in **Figure 2**. Furthermore, an ICOM-mediated interaction could also be involved in the formation of an CD19:CD79b/CD79b:CD19 complex that is found on the B cell surface after BCR internalization and can emit a pro-survival signal (52). In summary, the discovery of conserved ICOM sequences in the TMD of CD79b and several coreceptor modules suggests that the IgM-BCR regulation and activation is a dynamic process involving stable or transient ICOM-based homo- and hetero-dimerization of the IgM-BCR with itself and with CD45 and CD19.

### The ICOM interactome of T lymphocytes

Does this new insight into the ICOM-dependent regulation and activation of B cells process also have implications for the regulation of T cells? Given the prominent role of CD79b within the IgM-BCR complex, I asked whether the αβTCR has a similarly exposed ICOM-carrying signaling component. The cryo-EM structure of the αβTCR shows an 8-helix instead of the 4-helix TMD bundle of the BCR (18). The signaling subunits are organized as two heterodimers (CD3γ/CD3ε and CD3δ/CD3ε) and one TCR-ζ/TCR-ζ homodimer. Interestingly, the TMD AAs of the CD3γ and CD3δ chains establish different contacts within the asymmetric 8-helix bundle. All sides of the CD3γ TMD are involved in binding to either the CD3ε, the TCR-α or TCR-ζ TMD whereas the CD3δ TMD has a free side with an ICOM-related LZ motif (**Table 3**). It is thus likely that CD3δ either mediates TCR : TCR oligomerization (53, 54) or couples the αβTCR to lateral interactors such as the CD45/CD45-AP complex (8). Indeed, the ICOM-carrying side of the CD3δ TMD shares six identical or similar AAs with the CD45 TMD. Similar to the variant LZ motif of CD45, the human CD3δ LZ motif carries an isoleucine or leucine only at the three core positions, while the two outer positions are occupied by a threonine and a cysteine.

CD45 has been well studied as a gatekeeper of the resting state of T cells and the ICOM-based inhibitory CD3δ/CD45 complex may be a part of this important regulation. Another negative regulator of T cell activation is the transmembrane adaptor “phosphoprotein associated with glycosphingolipid-enriched microdomains 1 (PAG-1), also known as CSK binding protein (Cbp). PAG-1 is constitutively tyrosine phosphorylated in resting T cells and is associated with the C-terminal Src kinase CSK, which inhibits Src-family kinase (SFK) activity (55). It is only upon T cell activation that PAG-1 is dephosphorylated, allowing for the release of CSK and SFK inhibition. T cell activation is also under the control of the immune checkpoint inhibitor PD-1, which is targeted by anti-PD1 antibodies in several successful anti-tumor therapies. A recent study showed that the inhibitory function of PD-1 requires PAG-1 and that the two proteins are found in close proximity on the T cell membrane (56, 57). Genetic studies have also identified the scavenger receptor family member CD5 as a negative regulator of T cell selection and activation (58). Interestingly, all three of these proteins carry an ICOM-related LZ motif with a cysteine at the 5^th^ position (**Table 3**), and the CD3δ ICOM may help to couple these negative regulators to the αβTCR. T cell activation is accompanied by the recruitment of the “linker for activation of T cells” (LAT) to the TCR complex. The TMD of LAT also carries an ICOM-related LZ motif with a cysteine at the 5^th^ position (**Table 3**). Thus, it is possible that on activated T cells, LAT replaces PAG/PD-1/CD5 in its interaction with the CD3δ ICOM. Interestingly, it has been found that not only PAG-1 or LAT but also CD3δ can be palmitoylated on the cysteine at the 5^th^ position of the CD3δ ICOM (59). Such a palmitoylation prevents a S-S dimer formation but it does not prevent the Van der Waals interactions of the sulfur atom and thus may stabilize rather than compromise an ICOM-based interaction (46). The CD3δ ICOM may also recruit the adhesion protein CD44 and the T cell signaling modulator CD6 to the αβTCR. As described for the ICOM interactome of B cells, the ligand-dependent activation of T lymphocytes may involve a dynamic reorganization of ICOM-positive membrane proteins on the T cell surface (**Figure 2**). The suggestion that an ICOM interactome of TCR homodimers and heterodimers regulates the resting state of T lymphocytes and their transition into activation may solve a long-lasting controversy about the oligomeric or monomeric structure of the TCR on the surface of living T lymphocytes. Specifically, the ICOM discovery suggests that the experimentally found TCR monomers (60, 61) may actually be TCR : CD45, TCR : PAG or TCR : CD5 heterodimers and that TCR : TCR homodimers coexist with these heterodimers on different topographical regions of the same T cell surface.

## Discussion

Previously, it was thought that the TMDs only act as anchors for a membrane protein in the lipid bilayer. However, often a TMD sequence of a given membrane protein is evolutionary highly conserved, and this conservation suggests other important functions of a TMD besides anchoring. This has been impressively demonstrated by the recently published cryo-EM structures of the TCR and BCR complexes which revealed that the TMDs of the different receptor components occupy a considerable space and that specific TMD AA interactions play an important role in the stability and organization of the antigen receptor structure. Unfortunately, for most receptors, such as the EpoR or PDGFR, structural information is available only for the ectodomains but not for the TMD part, and this lack of information has resulted in misinterpretations of the receptor organization and function (29). For example, it was thought that most cell surface receptors are expressed as monomers that dimerize only upon ligand binding. This idea is no longer supported by more recent receptor studies. Many biochemical studies show that the TMD of single-spanning receptors form ligand-independent dimers or higher complexes in the lipid bilayer (24, 62). So far, two different TMD dimerization motifs have been studied, namely the GxxxG motif and the LZ motif (63). The GxxxG motif has been studied in the context of the glycophorin A function (64, 65) but it also plays an important role in the dimerization and proper membrane localization of murine and human MHC class II proteins (66, 67). The LZ motif has been extensively studied in the context of the EPO-R (68) and PDGFR (43). Interestingly, some of the ICOM-positive TMD sequences also contain a GxxxG motif and these TMDs may switch between different conformations. Compared to the GxxxG motif, the LZ motif is more flexible and can tolerate multiple AA exchanges. In particular, aliphatic and sulphur-containing AAs, such as methionine and cysteine, can increase the affinity of a TMD : TMD interaction (46, 69). This could be one of the reasons why many ICOM-positive membrane proteins on the T cell surface carry a cysteine at the 5^th^ position of the LZ structure.

The analysis described focuses on the TMD sequence of single-pass membrane proteins, but the TMDs of multi-pass membrane proteins may also carry an ICOM. However, without a detailed structural information it is not possible to determine whether the ICOMs connect the TMDs within the multi-pass receptor or establish a bridge to lateral interactors. An important class of multi-spanning membrane proteins are tetraspanins, which play an important role in the nanoscale organization of receptor clusters (70–72). Indeed, several members of this receptor family seem to carry an ICOM within their TMD (unpublished observation).

The recently determined cryo-EM structures of the monomeric TCR and BCR complexes were an important advance but they did not reveal the regulation and activation mechanism of these antigen receptors. The ICOM discovery suggests that CD3δ and CD79b are the main lateral interactors of the TCR and BCR, respectively. As outlined in **Figure 2**, these ICOM-positive receptor components could connect the BCR and TCR to negative or positive signaling coreceptors. I propose that antigen receptors do not function as isolated entities but rather as part of a dynamically regulated ICOM-based interactome that guards the resting state of lymphocytes and is remodeled during lymphocyte activation.

The events that lead to the dissociation and re-association of ICOM-positive receptor components are not clear at present. They may involve changes not only of protein interactions but also of the lipid composition. For example, upon antigen-induced opening of the CD79b/CD79b homodimer or CD79b/CD45 heterodimer the so far engaged AAs are exposed to the surrounding lipids and may change their composition in a way that favors dissociation and reorganization of the ICOM interactome. Furthermore, dynamic changes in the PIP2 versus PIP3 composition in the inner leaflet of the plasma membrane could have an impact on the ICOM-mediated interaction within the membrane (73). In summary, topological features of the plasma membrane, the dynamic colocalization of ICOM-positive receptors within specific lipid/protein domains and their closed or opened conformation may determine whether ICOM members interacte with each other on resting or activated lymphocytes. Furthermore, as discussed above, an established weak ICOM : ICOM interaction could be stabilized by extracellular (galectins) or intracellular (i.e. cytoskeletal components) crosslinkers. My hope is that further biochemical and genetic studies will confirm the role of the ICOM interactome and provide a solid foundation for a better understanding of adaptive immunity. These experimental ICOM studies may also promote the development of therapeutic peptides that increase or prevent an ICOM-mediated interaction within the membrane (74).

## Materials and methods

Human UniProt membrane protein sequences were downloaded (https://www.uniprot.org) and filtered for unique single-pass transmembrane proteins. The TMD sequence with 5 flanking N- and C-terminal AAs were extracted using the feature annotations for each gene. This resulted in 2417 unique single-pass TMD sequences, which were scanned for ICOM motifs. Motifs were formulated as regular expressions and matched using grep.

## Data availability statement

Publicly available datasets were analyzed in this study. This data can be found here: https://www.uniprot.org.

## Author contributions

MR: Formal Analysis, Investigation, Writing – original draft.
